# Therapeutic effect of microRNA-21 on differentially expressed hub genes in gastric cancer based on systems biology

**DOI:** 10.1038/s41598-023-49225-8

**Published:** 2023-12-11

**Authors:** Hesam Ghafouri Kalajahi, AmirHossein Yari, Mohammad Amini, Tunc Catal, Mahya Ahmadpour Youshanlui, Omid Pourbagherian, Cigdem Sezer Zhmurov, Ahad Mokhtarzadeh

**Affiliations:** 1 Department of Molecular Biology and Genetics, Uskudar University, Uskudar, 34662 Istanbul, Turkey; 2 Immunology Research Center, Tabriz University of Medical Sciences, Tabriz, Iran

**Keywords:** Biological techniques, Cancer, Cell biology, Computational biology and bioinformatics, Molecular biology, Systems biology, Gastroenterology

## Abstract

Gastric cancer (GC) is a leading cause of mortality for many people. Cancer’s initiating factors are poorly understood. miR-21 has a crucial function in several malignancies, particularly GC. Furthermore, it has been shown that miR-21 is critical for the emergence and advancement of GC. This work intends to identify new genes which expression is associated with the activity of mir-21 in GC and to investigate the effect of downregulation of mir-21 on these genes and gastric tumorigenesis. We utilized the gene expression profiles of GCs from an Array database (GSE13911) from the Gene Expression Omnibus (GEO) dataset to find differentially expressed genes (DEGs) between control and gastric cancer groups. Using weighted gene correlation network analysis (WGCNA) in R, the Gene co-expression network was reconstructed. The microRNA–mRNA network was then reconstructed using the miRWalk database, and by investigating the microRNA–mRNA network, the genes that have an association with mir-21 were found. To implement the functional investigation, MKN and AGS cell lines were transfected with anti-miR-21 next. Subsequently, MTT proliferation was utilized to assess the cell's vitality. qRT-PCR was then used to evaluate the anticipated levels of gene expression in both GC cell lines. This study discovered and predicted CCL28, NR3C2, and SNYPO2 as the targets of miR-21 (GC), which are downregulated through gastric tumorigenesis, showing great potential as therapeutic and diagnostic targets. The suppression of miR-21 in gastric GC cells led to the inhibition of cell proliferation and decreased expression of CCL28, NR3C2, and SNYPO2 genes. This study established that miR-21, via downregulating these genes, contributes significantly to the development of GC. In addition, systems biology techniques identified CCL28, NR3C2, and SNYPO2 genes as possible GC surveillance and therapy components.

## Introduction

Gastric cancer (GC) is one of the most widespread cancers worldwide and the 2nd most common reason for cancer demise globally^1^. One of the important problems in GC therapy is that the majority of y GC cases are detected with progressive-step disease because of the absence of appropriate biomarkers^2^. Therefore, new biomarkers are critically required for enhancing GC, initial detection, prognostic assessment, and tumor classification^3^. In addition, the effectiveness of traditional cancer including surgery and radiotherapy for advanced GC is not sufficiently good, and cancer development and metastasis are the main causes of the unacceptable survivorship of GC patients in the progressive step. Consequently, it will be important for GC therapy that determines the molecular pathways for the growth and advance of GC and detected remedial objectives^4,5^.

MicroRNAs (miRNAs) are a novel group of small noncoding RNA (ncRNA) that controls the up and down-regulation of different genes via translational suppression or mRNA slicing^6^. It has been shown that miRNAs intercede varied physiological activity, including cell growth, reproduction, programmed cell death, and metabolism process^7^. Current investigations support the important function of miRNAs in the proliferation, development, and progression of a diversity of cancer, particularly GC^8^. MiRNAs are involved in GC and display action as both tumor inhibitors and oncogenes (oncomiR)^9,10^. miR-21 is commonly upregulation in a different type of cancers, such as grade IV astrocytoma, breast cancer, oral cancer, ovarian cancer, hepatoma, cervical cancer, NSCLC, and GC. In vitro investigations in tumor cell cultures reveal that miR-21 have an essential function in the oncogenic procedure, including increased proliferation, invasion, and metastatic and decrease apoptosis capability^6,11,12^. miR-21 is upregulated in GC and its abnormal expression may have an essential function in GC development and diffusion via up downregulating of the antioncogenes *PTEN* and *PDCD4*, also via regulating the pathways relate to interceding cell development, migration, metastasis and programmed cell death^13,14^. Though an enhanced expression rate of miR-21 has been detected in GC, researchers associated with the function of miR-21 in GC development is extremely restricted^15^.

Newly, with the assistance of microarray and RNA‐sequencing method, also public datasets including Gene Expression Omnibus (GEO) and The Cancer Genome Atlas (TCGA), several gene expression investigations on GC have been registered in the recent years. But because of the restricted sample amounts and unsuitable analysis techniques, mistakes, noises also outliers of differentially expressed genes (DEGs) outcomes may be attained^16,17^. Regarding this problem, the weighted correlation network analysis (WGCNA) technique became one strong method to attain considerable DEGs. This network analyzer is used for determining the gene or miRNA clusters related to the advance of several malignancy and the new possible detection tool associated with early diagnosis^18^.

**Figure 1:**
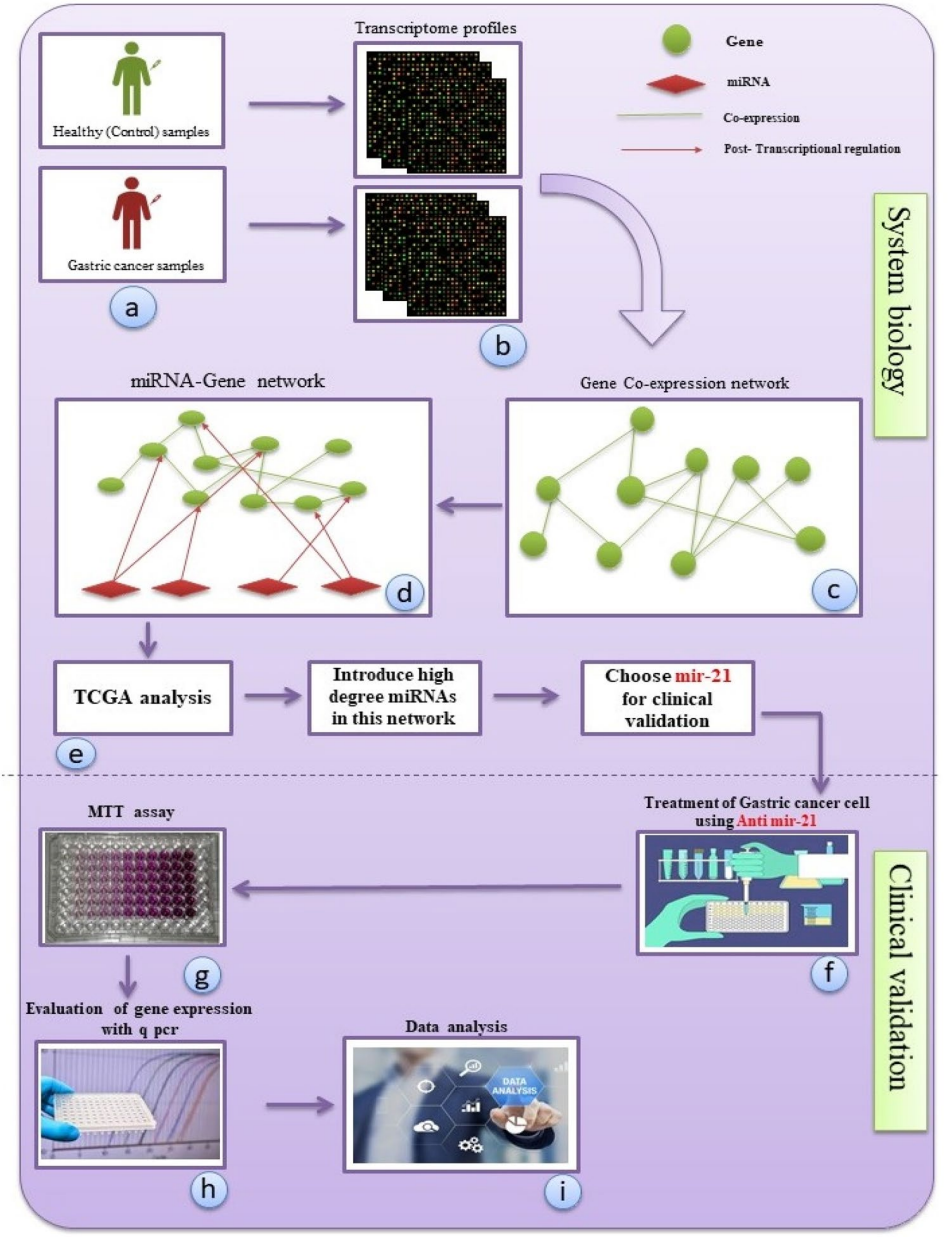
The flow chart and overview of this research.

In this investigation Using bioinformatics, we looked for genes with differential expression between GC and normal tissues. GEO2R was used to normalize and preprocess the unprocessed data. Using DEGs with an adj p-value less than 0.01 and LogFC less than − 1, the co-expression network was created. Reconstruction of the co-expression network using the WGCNA package in R 4.1.2 To generate co-expression modules, the expression levels of 1720 genes were inputted into WGCNA. The relative expression level of miR-21 was determined by qRT-PCR using ExiLent SYBR Green master mix and specific primers for miR-21. Following that, cells were transfected with anti-miR-21 and incubated. On the basis of system biology, we finally identified the therapeutic impact of anti-miR-21 on DEGs in GC. As a result, the diagnosis of efficient genes associating with GC and the detection of their correlation with mir-21 using molecular techniques would not only be rapid but also cost-effective, and it would allow for the early detection of cancer as well as the possibility of less treatment and more effective outcomes (Fig. 1).

## Results

### Dataset and data processing

To discover genes associated in gastric cancer, we analyzed the GSE13911 microarray dataset. The characteristics of this dataset are detailed in Table 1. This dataset included 31 normal tissue samples and 38 stomach tumor tissue biopsies.

**Table 1:** GSE9348's dataset details.

Groups	Source name	Number of samples	Expression array
Normal samples (healthy control samples)	Adjacent normal tissue	31 samples	GPL570: [HG-U133_Plus_2] affymetrix human genome U133 Plus 2.0 array
Gastric cancer	Tumor biopsy	38 samples	

**Figure 2:**
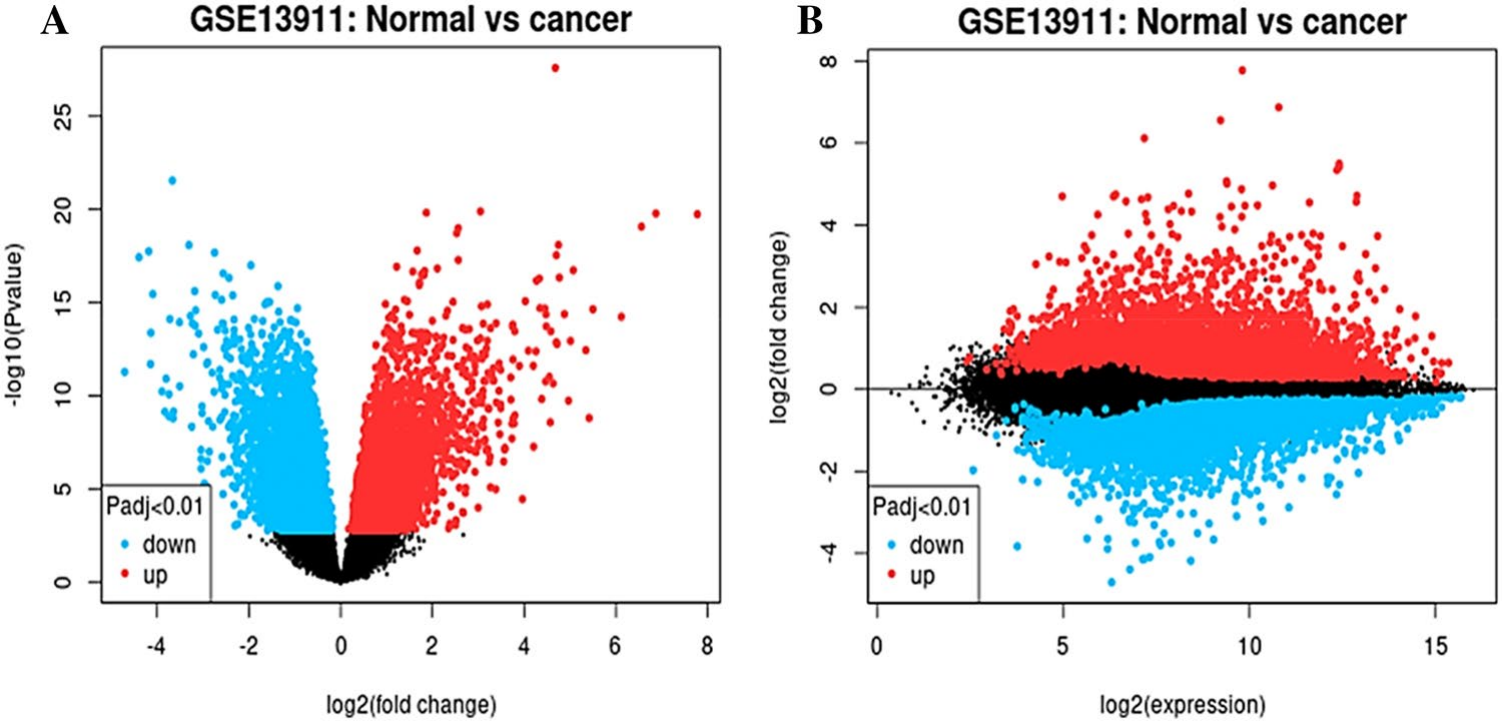
Volcano plot (**A**) and mean-difference plot (**B**) of dataset.

Comparing gastric cancer samples to normal samples revealed 54,675 genes with differential expression. 1720 genes with adj p-values less than 0.01 and fold changes less than − 1 were identified as differentially expressed (Fig. 2).

### Weighted gene correlation network analysis (WGCNA) and module preservation analysis

**Figure 3:**
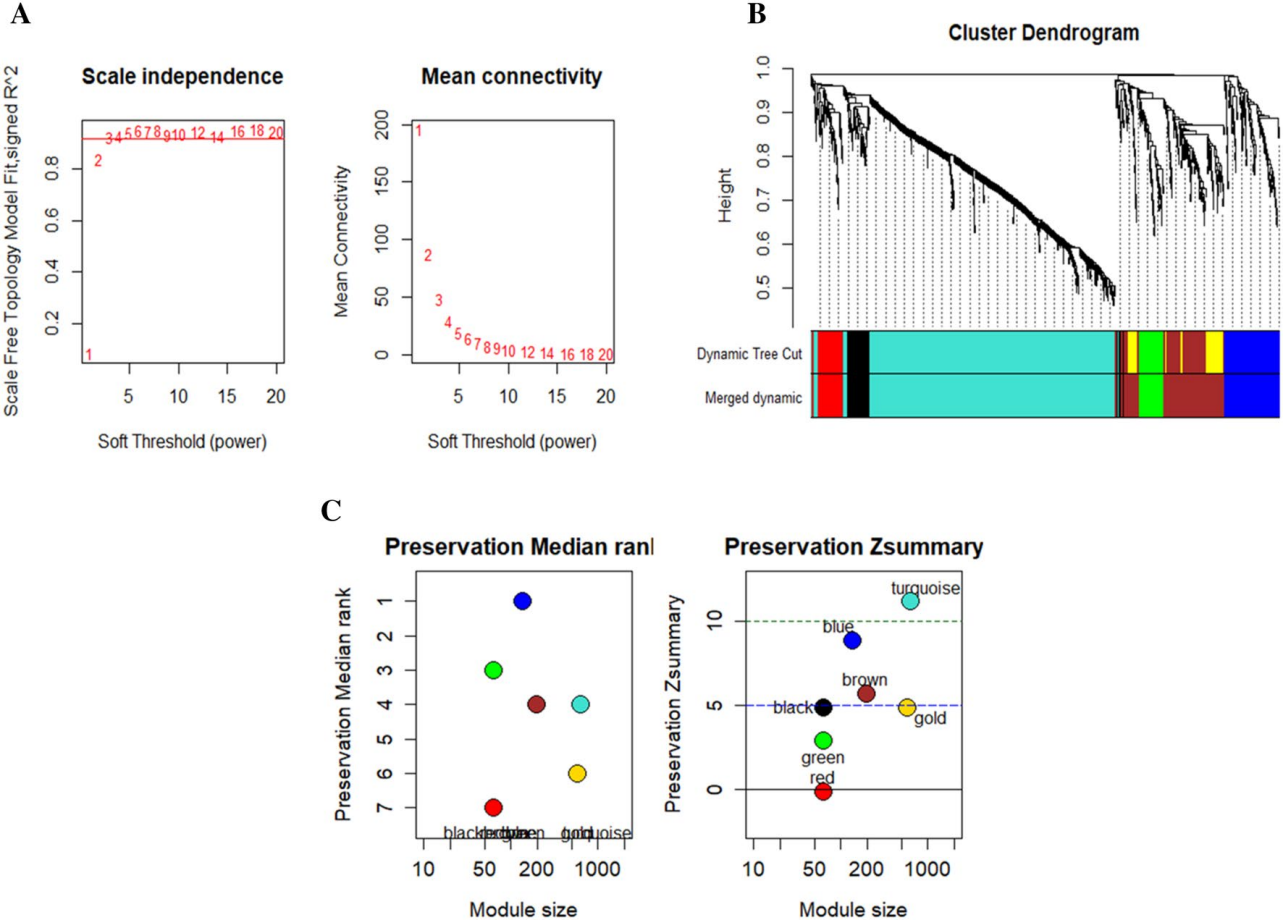
(**A**) R2 demonstrates how the scale independence of co-expression of modules changes based on the power value and Mean connectivity for soft-threshold power levels in the co-expression network. (**B**) Co-expression of genes is being investigated. Analysis of gene expression networks in British Columbia has shown distinct modules of co-expressed genes. The vertical axis represents the value of gene expression, while the horizontal axis represents the number of genes in the sample. In the dendrogram, each vertical line represents a gene, and each branch indicates a module of highly co-expressed genes (one color). Modules that have been detected appear in the first color band, whereas modules that have been combined appear in the second. (**C**) Module preservation diagram with Z summary.

Data from 31 normal tissue samples and 38 biopsy samples of gastric tumor tissue were utilized to construct the co-expression network. As the optimal value for the network's scale independence, WGCNA had an R2 of 0.8550 and greater average connectivity with screen-out powers of 1 to 20 and a topology index (R2) of 10. Expression data matrices were created after that. In other words, we utilized it to compute our matrix of topological overlaps (TOM) (Fig. 3). Each module's Z-score was summarized using the Module Preservation function. We discovered 2 modules using Zsummery2. These modules are green and red, and the green module was analyzed further.

### MicroRNA–mRNA network

**Figure 4:**
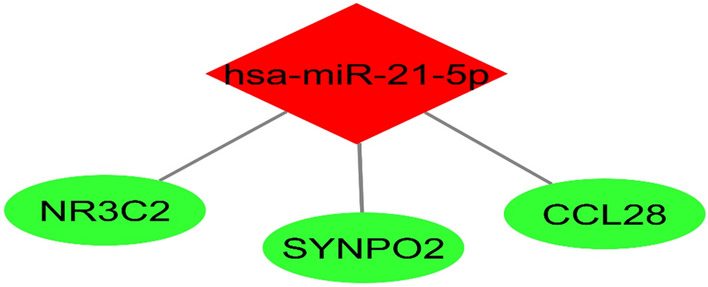
microRNA–mRNA network and target genes of mir-21. Green ellipses represent genes and red diamond represent microRNA.

Using the miRWalk database, microRNAs that target green module genes were identified. The microRNA–mRNA interactions of modules' genes were then determined. Due to the substantial overexpression of mir-21 in gastric cancer, its target genes were identified from the network and visualized using Cytoscape V3.9.1, an open-source software platform for visualizing complex networks and integrating these with any type of attribute data (https://cytoscape.org/) (Fig. 4).

### TCGA validation

**Figure 5:**
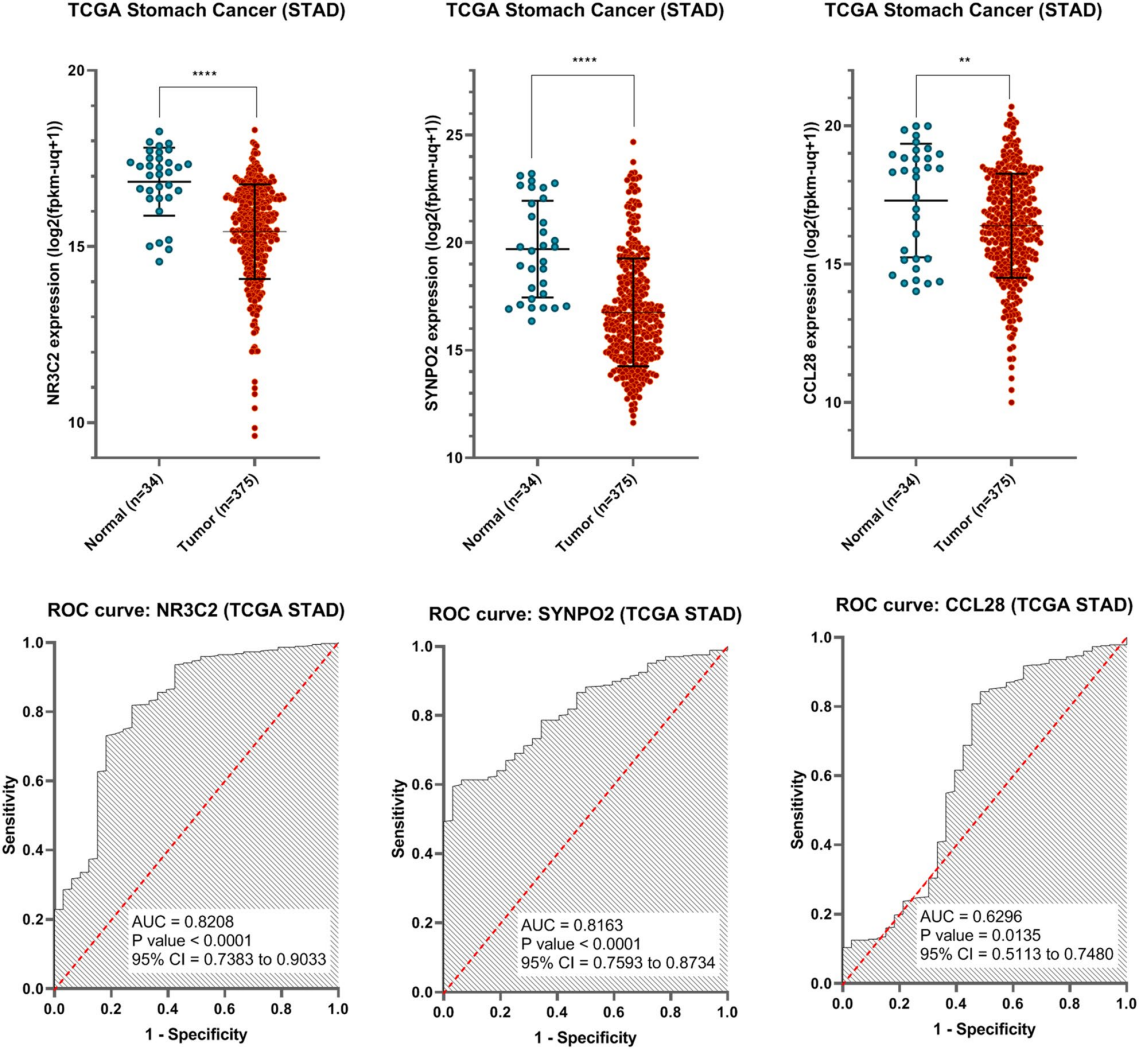
Expressions of NR3C2, SYNPO2, and CCL28 may provide as diagnostic markers for GC cancer and normal samples according to TCGA-STAD.

GEO datasets and a co-expression network were also validated using the TCGA database. NR3C2, SYNPO2, and CCL28, three genes targeted by mir-21, were shown to be highly expressed in both stomach cancer and healthy tissues. In comparison to normal samples, TCGA data show a substantial reduction in the expression of these genes. For GC and normal samples, the AUC of NR3C2, SYNPO2, and CCL28 may be used as diagnostic indicators, as illustrated in Fig. 5. These genes' p-values, AUCs, and biomarker abilities are shown in Fig. 5

### Tissue validation

**Figure 6:**
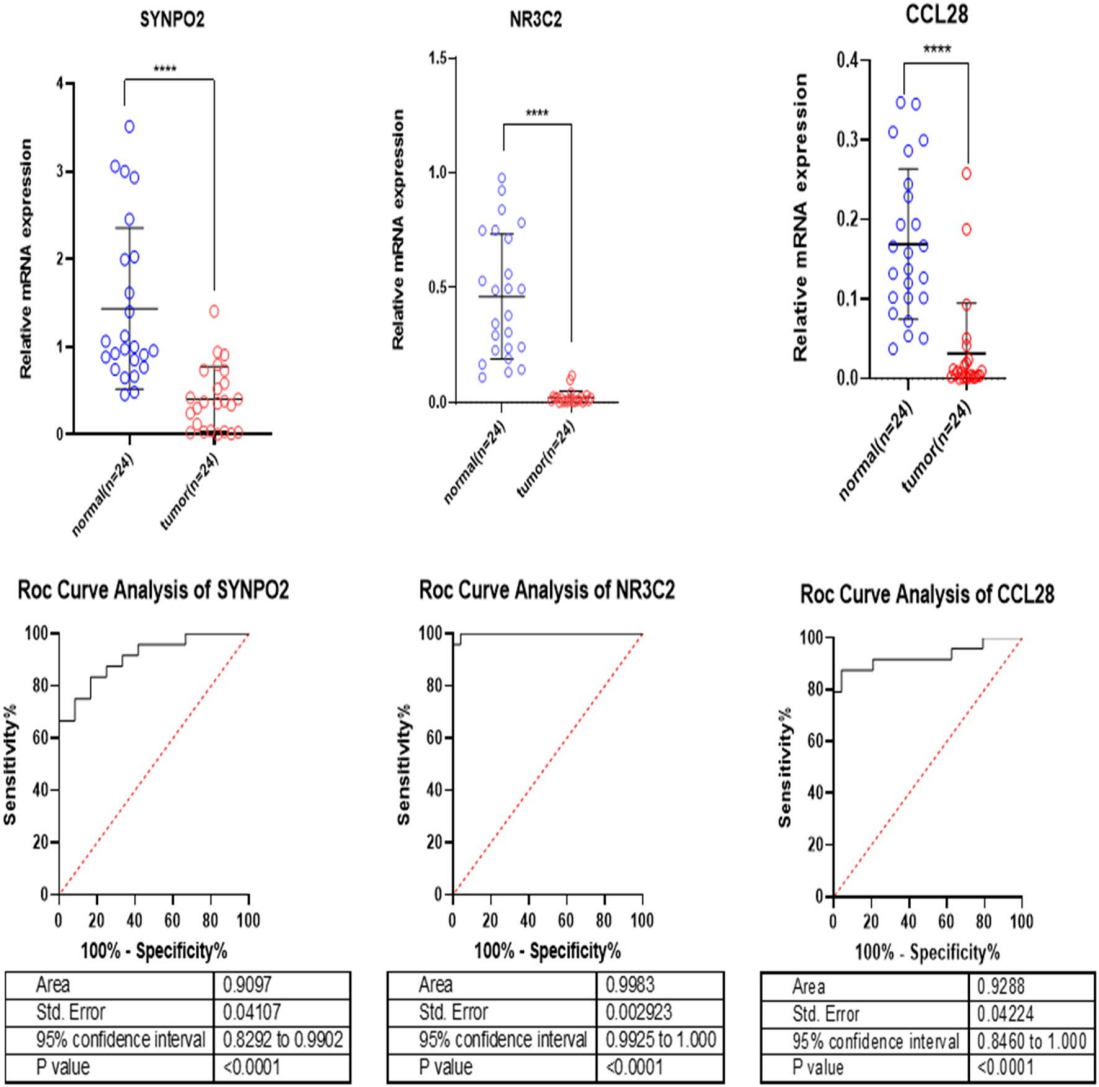
The potential diagnostic use of NR3C2, SYNPO2, and CCL28 expressions in distinguishing between gastric cancer (GC) and normal tissue samples is worth considering.

Quantitative polymerase chain reaction (qPCR) was used to examine the expression levels of SYNPO2, NR3C2, and CCL28 in a total of 24 gastric cancer samples and 24 margin samples. The examination of quantitative polymerase chain reaction (qPCR) data demonstrated a substantial downregulation (p value < 0.0001) in the expression of SYNPO2, NR3C2, and CCL28 in gastric cancer tissue samples when compared to matched tumour margin samples (Fig. 6). These findings corroborate the results obtained from The Cancer Genome Atlas (TCGA) data. The results of rock curve analysis demonstrated that the differential expression of SYNPO2, NR3C2, and CCL28 had a major role in distinguishing tumour tissues from borderline samples (Fig. 6).

### miR-21 suppression in GC cell lines

**Figure 7:**
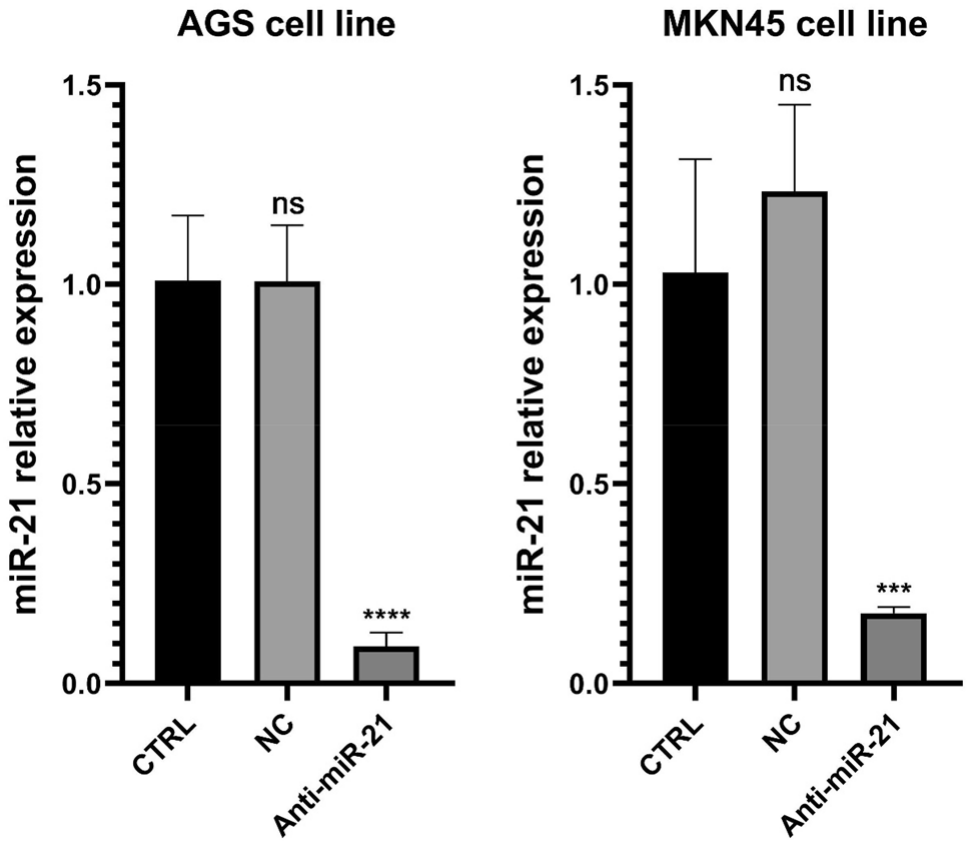
The effect of anti-miR-21 on miR-21 expression levels in AGS and MKN45 cell lines. miR-21 relative expression level was recognized via qRT-PCR, and all statistics were normalized via U6 RNA. ****Adjusted p value less than 0.0001. ***Adjusted p value less than 0.0005.

The qRT-PCR was applied to evaluate the miR-21 expression levels in AGS and MKN45 cell lines. The results obtained from qRT-PCR evidenced that transfection of GC cells with anti-miR-21 led to the downregulation of this miRNA. As shown in Fig. 7, miR-21 expression in transfected AGS and MKN45 cells was significantly reduced compared to negative control (NC) and control groups.

### The effect of miR-21 suppression on GC cell proliferation

**Figure 8:**
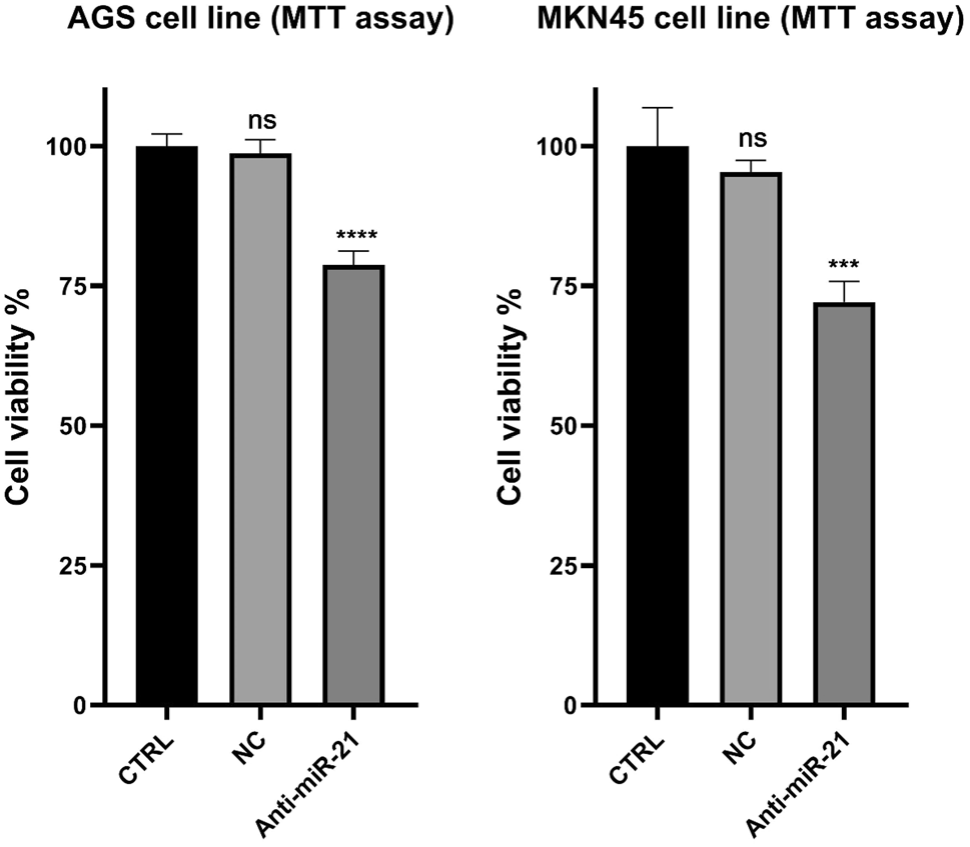
Combination efficacy of Anti-miR-21 on the viability of AGS and MKN-45 cells. Then their viability was assessed through MTT test. ***p < 0.0001, ****p < 0.0006.

To investigate the effect of miR-21 suppression on GC cell proliferation, MTT assay was employed (Fig. 8). According to the results, the survival rate of AGS and MKN45 cell lines significantly decreased after transfection with anti-miR-21.

### The efficacy of miR-21 on the rate of *CCL28*, *NR3C2*, and *SYNPO2* hub gene expressions in AGS and MNK45 cells line

**Figure 9:**
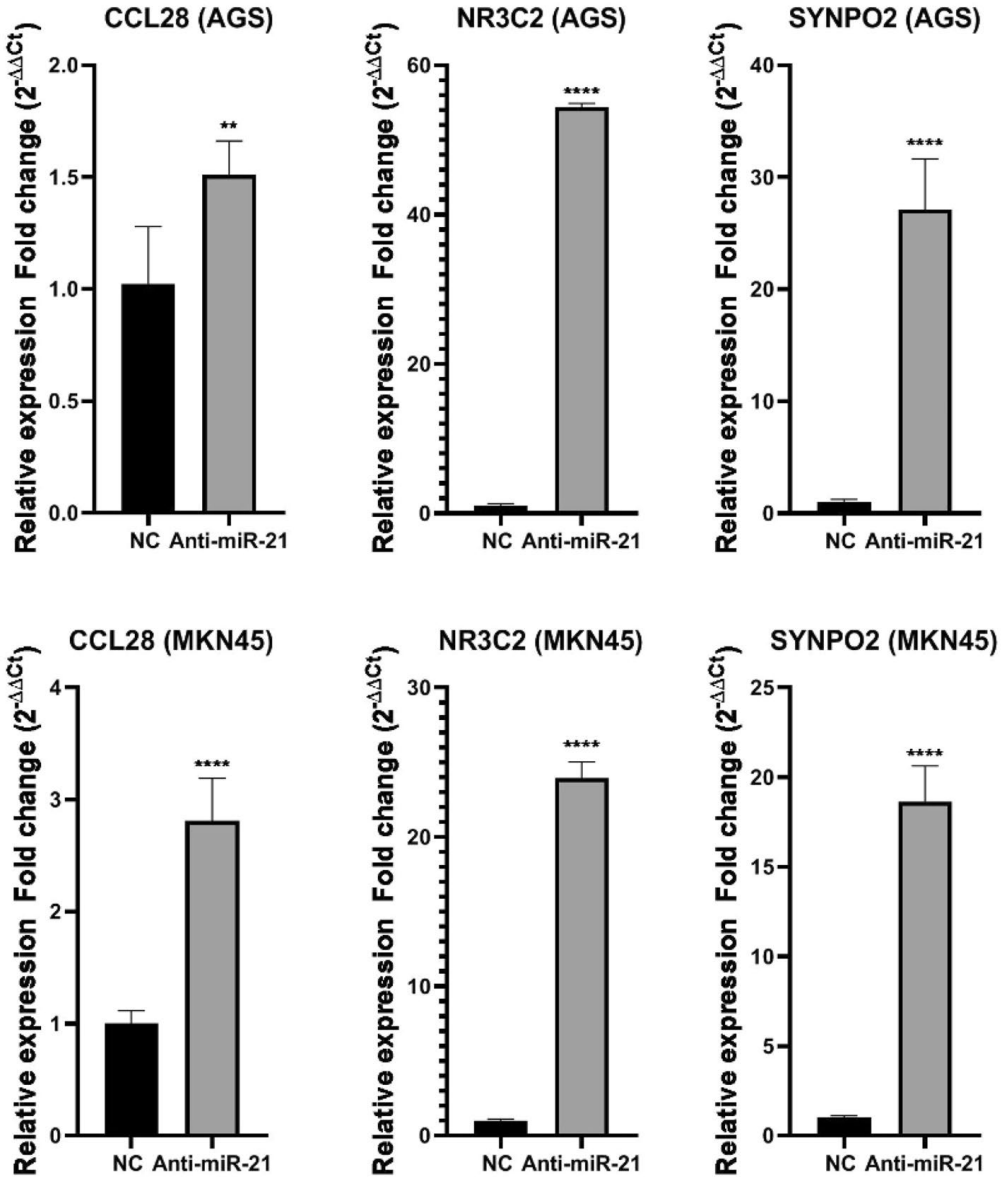
The qRT-PCR analysis of *CCL28*, *NR3C2*, and *SYNPO2* gene expression was done in the non-transfection and Anti-miR-21. Anti-miR-21 increased genes expression in both cell lines. **p < 0.0084, ****p < 0.0001.

qRT-PCR was applied to evaluate the *CCL28*, *NR3C2*, and *SYNPO2* gene expression levels in AGS and MKN45 cells. Here of, afterward the transfecting of AGS and MKN45 with Anti-miR-21, the outcomes displayed that CCL28, NR3C2, and SYNPO2 genes expressions were considerably increased in the both cell lines (Fig. 9).

## Discussion

**Figure 10:**
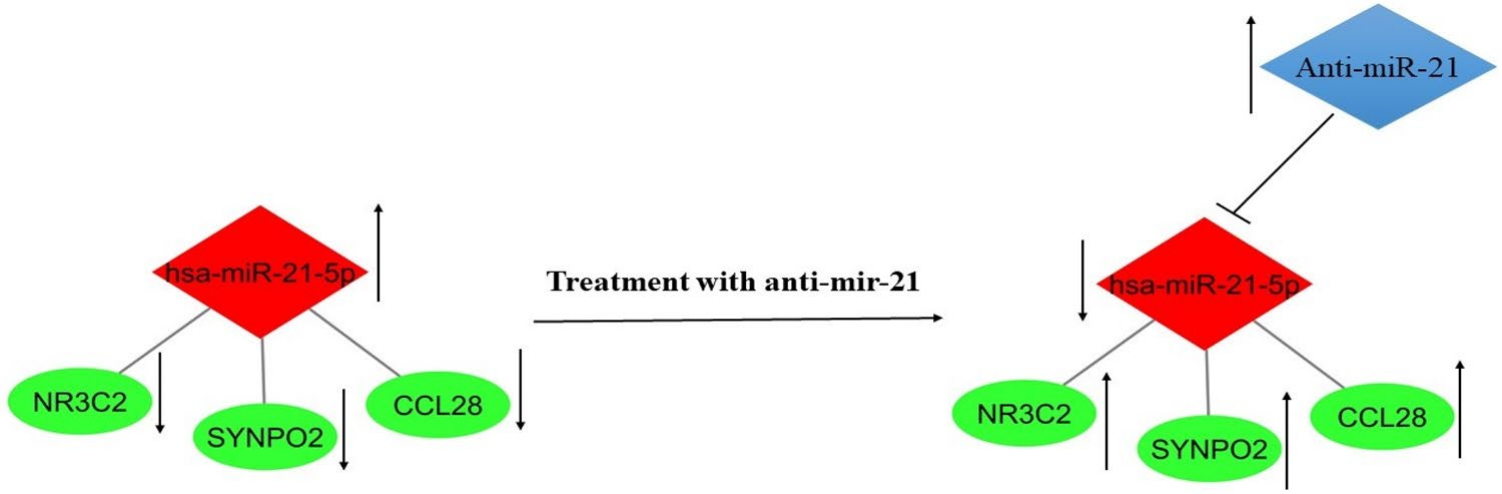
Therapeutic effect of miR-21 suppression on hub genes.

This study aims to identify the target genes of mir-21 for diagnostic and therapeutic applications. Consequently, bioinformatic analyses were used to explore for genes that are differently expressed in GC tissues vs normal tissues. In addition, the co-expression network was rebuilt using DEGs with an adj p-value of less than 0.01 and a logFC of less than − 1. The co-expression network was rebuilt using R 4.1.2's WGCNA package. In order to generate co-expression modules, the expression levels of 1720 genes were input into WGCNA. The relative expression level of miR-21 was determined by qRT-PCR using ExiLent SYBR Green master mix and specific primers for miR-21. Following this, cells were transfected with anti-miR-21 and incubated. Lastly, we assessed the therapeutic impact of anti-miR-21 on DEGs in GC using system biology. As a result, the diagnosis of efficient genes associating with GC and the detection of their correlation with mir-21 using molecular techniques would not only be rapid but also cost-effective, and it would allow for the early detection of cancer, as well as the possibility of a shorter treatment process and more efficient outcomes. The results of this study indicate that overexpression of mir-21 has a negative effect on the expression of CCL28, NR3C2, and SYNPO2 and plays a significant role in the progression of gastric cancer, whereas suppression of mir-21 with anti-mir-21 has an effect on the expression of CCL28, NR3C2, and SYNPO2. (Fig. 10).

GC shows a significant participant in the worldwide burden of cancer^19^. Factors that increase the risk of GC include: Helicobacter pylori infection, genetic, age, gender, nationality, nutrition, and smoking^20,21^. GC patients display “3 high and 3 low” features, whereby the occurrence, stages of metastasis, and fatality percentage are great, and the initial detection percentage, radical elimination amount, and 5-year survival percentage are less^22^. Thus, it is especially significant to advance surgical therapy selections for progressive GC. There has been novel advance in improving therapeutic method, and augmented comprehension of the pathogenesis of GC has led to methods to the inhibition of GC, and the advance of targeted treatments. But, despite the fast expansion of radiotherapy, chemotherapy, and immunotherapy, surgical resection remains the only potential treatment for GC^23^. In spite of progresses in multimodality cure, the recurrence amounts is still high^24^. Contrasted with other cancer, there is an absence of simple to assess biomarkers for gastric cancer, which is related on an unfavorable clinical result in many cases^25^. Therefore, while providing an improved comprehension of the controlling pathways is done via these molecules, the study of such epigenetic bases may as well as result in the detection of novel biomarkers that are adequately accurate and particular, so improving aspects associated with late detection and response to therapy^26^. Different molecules as detection tools are an effective method to early diagnosis of cancer and for improving targeting treatment. MicroRNAs (miRNAs) expression influences the development, growth, and triggering of immune cells and tumor immune reaction, so resulting in protumor or tumor suppression activities. As regulating short noncoding RNAs, miRNAs are engaged in many cellular and molecular processes. They inhibit gene expression and modulate various signaling pathways, which contributes to cancer. Thus, these compounds may represent potential biomarkers and therapeutic targets for better cancer treatment for example a research shows miR-81's significance in many cancers^27^. Therapeutic medicines may potentially find miRNAs (micro RNAs) to be useful targets. Significant potential for miRNAs as a therapeutic agent in combination cancer therapies MicroRNA-193a and taxol were shown to have synergistic effects in colorectal cancer^28^. Therefore, miRNAs have an anti-cancer role can be possible agents for the advance of novel treatments. Between the different miRNAs, the change of miR-21 expression in cancer tissues is increasingly offered as a reliable predictive biomarker of survival for GC^29–31^. A new study has shown that the noncoding RNA mir-21 plays a significant role in the development of stomach cancer. To establish this correlation, the researchers conducted molecular and cellular tests and the research findings indicate that the use of anti-miR-21 exhibits potential as a viable therapeutic strategy for the treatment of gastric cancer (GC)^32^. Research found that miR-21 is overexpressed and functions as an oncomiR in many human cancers by targeting tumor suppressor genes involved in apoptosis, proliferation, and metastasis. In particular, miR-21 causes drug resistance and its overexpression is linked to breast cancer MDR^33^. In a novel investigation, Janson Tse et al. displayed that miR-21 expression in a preclinical sample of initial GC is affiliated with Stat3 downstream of the IL-6 family cytokine-interceded triggering of gp130 receptor signaling. Anti-miR-21 treatment inhibited GC tumor development, and limited epithelial-to-mesenchymal transmission and matrix remodeling^34^. In another investigation, researchers evaluated the expression rates of plasma circulating oncogenic miR-21 and miR-192 and their dependence on clinical phenotypes of patients with GC in the north of Iran. The expression amounts of miR-21 and miR-192 were assessed through qRT-PCR in the plasma of twenty patients with GC before and after surgery and 20 normal individuals. This investigation showed that plasma miR-21 expression was considerably related on tumor step and helicobacter pylori infection situation. But no dependence was detected among clinic-pathological features and miR-192 expression. The outcomes displayed that the plasma rates of miR-21 and miR-192 were considerably upper in GC patients compared to those in normal groups^35^. Moreover, investigators showed that exosomal miR-21-5p lead to increase GC via triggering TGF-β/Smad pathway via targeting SMAD7. As result, exosomal miR-21-5p stimulates MMT of PMCs and increase cancer peritoneal diffusion via targeting SMAD7. The exosomal miR-21-5p may be a new remedial agent for GC peritoneal metastasis^36^. Furthermore, inhibit of miR-21 considerably reduced cell invasion and migration of AGS cell lines. Also, RECK, recognized as a tumor suppressor in GC, is an important target of miR-21. Overall, miR-21 may be vital in the beginning and advancement of GC as an oncomiR, probable by regulating RECK. In addition, *H. pylori* infection increases expression of miR-21, which reduces RECK expression, and next results in the progress of GC^6^. Furthermore, miR‑21‑5p was considerably upregulated in GC and negatively related on PDHA1 expression in GC^37^. Expression of miR-21 led to downregulation T-AOC, SOD and CAT; however, the expression level of late 8-OHdG and hOGG1mRNA was increased. Moreover, increased expression of miR-21 leads to decreased expression of PDCD4^38^. Generally, miR-21 is upregulated in GC and its abnormal expression may have essential function in GC development and spreading by regulating the expression of the PTEN and PDCD4, also via regulating the pathways involved in interceding cell development, migration, metastasis and apoptosis. Targeting miR-21 may assistance progress new treatment methods for GC^13^.

In recent studies, researchers have been studied on DEGs in both GC and healthy tissues and they found out the 7 of 10 hub genes are related on poor cancer prognosis by bioinformatics search and it would provide new insight for diagnosis and molecular mechanism and treatment of GC in early stages^39^. on the other hand, another research identified 295 DEGs of GC, and 7 genes are confirmed and considered as hub genes in GC, which are potential both for predicting and therapy of GC^40^. In this research, it was identified that the Anti-miR-21 reduced GC cell viability. Furthermore, the *CCL28*, *NR3C2*, and *SYNPO2* hub gene expressions in the cells transfected with the Anti-miR-21 were significantly enhanced in AGS and MKN45 cell lines. Pursuant to the previous research’s findings and the present investigation, we showed that Anti-miR-21 enforces anti-cancer efficacy via particular upregulation of hub genes, include *CCL28*, *NR3C2*, and *SYNPO2*. These results may simplify the further method for finding new therapeutic approaches in GC.

## Conclusion

Due to the fact that a suitable biomarker has not yet been found for initial diagnosis of GC and the methods used today are very expensive and invasive, there is a need for further studies in this field. So, by the diagnosis of efficient genes as biomarkers in relating on GC and detecting the correlation of them with mir-21 by simple molecular techniques, not only it would be rapid but also it would be economic and it will provide detecting of cancer in early stage, and it would give an opportunity of less treatment process and most effective results. Furthermore, this study demonstrated that overexpression of mir-21 has a negative effect on the expression of CCL28, NR3C2, and SYNPO2 and plays a significant role in the progression of gastric cancer, whereas suppression of mir-21 with anti-mir-21 has an effect on the expression of CCL28, NR3C2, and SYNPO2.

## Materials and methods

### Datasets and data processing

The following search terms were used to get gene profiles from the GEO database: (1) stomach cancer, (2) gastric cancer, and (3) gastric tumor biopsy. Then validated the following dataset: GSE13911 (https://www.ncbi.nlm.nih.gov/geo). GSE13911 included 31 normal tissue samples and 38 stomach tumor tissue biopsy samples. To normalize and preprocess the raw data, GEO2R was used. Differentially expressed genes (DEGs) have been selected for further analysis. By comparing the expression patterns of 31 normal tissues with those of 38 stomach tumor tissue samples, we were able to identify these DEGs. GEO2R, an online tool that employs many R packages to compute metrics including LogFC, p-value, and modified p-value, was used to conduct the comparison. After accumulating the differentially expressed genes, we subjected them to a selection criterion prior to running the WGCNA (Weighted Gene Co-expression Network Analysis). Those genes were selected for further study that had an adjusted p-value of less than 0.01 and a LogFC (logarithm of fold change) value of more than − 1. GEO2R, which was provided by the National Center for Biotechnology Information (NCBI), was used to identify DEGs among two linked sets^41^. GEO2R uses the GEO inquiry and R utilities from the Bioconductor package to impose analogies on main submitter-supplied processed data tables^42^.

### Weighted gene correlation network analysis (WGCNA)

The co-expression network was created by utilizing DEGs with an adj p-value less than 0.01 and fold change less than − 1. Using the WGCNA package in R 4.1.2. The network was created by the R package "WGCNA." The expression levels of 1720 genes were entered into WGCNA with the purpose of constructing co-expression modules. An algorithm called WGCNA was employed to determine gene expression levels. Using the flashClust tool in the R environment, cluster analysis was utilized to discover data with outliers^43^. After no further grouping of the outlier data, Descriptive and inferential statistics between all differentially expressed genes were calculated. In order to reconstruct the network for genes with strong connections and exclude those with weak ones, a parameter called β (soft thresholding power) was developed depending on the size of the network. In other words, scale-free topology^44^. A topological overlap matrix (TOM) and dissimilarity measure were used to identify the modules. Hierarchical clustering was used to create gene dendrograms, and the dynamic tree-cut approach was used to identify modules of co-expressed genes as branches of the gene dendrogram^45^. DeepSplit was set to 3, and the minimum module size was 50. Combining modules with comparable gene expression patterns is possible because of their proximity. As the first and most crucial element in each module, A gene expression profile is represented by the module eigengene, which serves as a summary for each module. This was followed by the creation of a series of subgroups based on how closely they were related. The dissimilarity between the modules and the highly expressed modules was fixed at 0.14. Module-trait relationships were used to quantify the connections between the modules and each early-stage cervical cancer subtype (MTR)^46^.

### Module preservation

"Module Preservation" was used to find modules shared across the two datasets. In addition, the Z-score was summarized using this method. The Z_Summary_ score is what we used to evaluate module preservation in this investigation. No conservation, poor to moderate preservation, or important preservation are considered for modules with Z_summary_ values less than 2, 2–10, or higher, respectively^47^. Modules are having a "Z_summary_" value less than 2 were the ones we selected. The Z score summary is a key measure in the WGCNA R program, which evaluates gene expression. It evaluates how much a gene's expression deviates from the mean in a certain tissue or illness situation. For the Z score, the observed gene expression value is subtracted by its mean expression value and divided by the standard deviation of expression across all samples. Normalization makes gene expression data comparable. Z scores provide researchers a standardized measure of how much a gene's expression varies from the context's average. Z scores in the positive or negative range imply above-average or below-average expressiveness. Larger Z scores indicate greater variations from the mean expression. Researchers may select genes with extremely significant expression changes and prioritize them for future study using WGCNA's Z score report. This measure helps identify genes' functional relevance in a co-expression network and define the biological processes involved in various contexts or circumstances^44,46,48^.

### MicroRNA–mRNA network

We combined to create a microRNA–mRNA network after identifying the most impressive mRNAs. For this purpose, the miRWalk database was used. The Cytoscape software was used to visualize the network. Due to the significant overexpression of mir-21 in gastric cancer the mir-21 and target genes were selected for further investigations.

### TCGA validation

RNAseq gene expression data were obtained and interpreted using the UCSC Xena Functional Genomics Explorer )https://xenabrowser.net/). There was also an evaluation of possible target genes for the mir-21 expression pattern in stomach cancer using the TCGA, which was done using a receiver operating characteristic (ROC) curve study. Based on the expression of these genes in tumor and normal tissue samples received from TCGA-STAD, patient and control values were determined. The ROC curve was generated and evaluated in GraphPad Prism, where p-values and the AUC were also calculated.

### Tissue validation

In order to confirm the expression data acquired from the Cancer Genome Atlas for gastric cancer (TCGA-STAD), a quantitative polymerase chain reaction (qPCR) technique was used on a total of 24 gastric cancer tissue samples and 24 normal samples. The research study received approval from the Ethical Committee of Tabriz University of Medical Sciences located in Tabriz, Iran. Prior to participating in the study, all patients provided written informed consent, explicitly granting permission for the publication of their data. The authors affirm that all procedures were conducted in compliance with the applicable guidelines and regulations. In order to achieve the objective, stomach cancer tissue samples were procured from patients. Prior to the surgical procedure, none of the patients had any kind of chemotherapy or radiation treatment. The samples were subjected to freezing using liquid nitrogen and thereafter preserved at a temperature of − 80 °C until the extraction of RNA was performed. Following the technique, Trizol (Gene All Brand Riboex) was used to extract total RNA. RNA quantity and quality were determined using NanoDrop spectrophotometer. The PrimeScriptTM RT Reagent Kit (TaKaRa Bio, Japan) was used to synthesise cDNA from 1 µg of total RNA in 20 µL using the standard procedure (50 °C for 30 min, 95 °C for 5 min, 10 °C for end) in the thermocycler PCR. The BioFACT™ 2 × Real-Time PCR Master Mix and gene-specific primers were used for Real Time PCR. The total reaction volume was 10 µL. Three measures were taken to react: Holding at 95 °C for 13 min is step 1. Step 2: 45 cycles of denaturation at 95 °C for 10 s, primer annealing at 60 °C for 30 s, and extension at 72 °C for 20 s. Step 3: melting curves were produced after each run.

### Cell culture

AGS and MKN45 cell lines was provided from the Pasteur Institute of Iran (IPI) and cultured in RPMI medium comprising 10% fetal bovine serum (FBS). After that, cells were incubated in distinct flasks in a moistened atmosphere of 95% comprising 5% CO_2_ at 37 °C. The cells were passaged at a primary concentration of 5 × 10^5^ Cells/ml and utilized in all experiments in the log phase of growth.

### Anti-miR-21 transfection

Anti-miR-21(5′-UCAACAUCAGUCUGAUAAGCUA-3′) was transfected via electroporation technique according to the company's instructions (about 500 ml electroporation buffer in each cuvette). After that, it was suitable amount of neutralizing medium and 200 μL of cell suspension and complete medium containing 12 × 10^3^ cells in each well were distributed from 96-well plates. The plate was included in a 37 °C incubator for 24 h to adhere the cells to the floor of the plate.

### MTT assay

To viability assessment, the cells were divided into 3 groups: control group (non-transfected cells), negative control (NC) and Anti-miR-21 group. After the cells reached the desired frequency, the cells were separated from the bottom of the flask. 200 μL of cell suspension and complete medium containing 12 × 10^3^ cells were distributed into 96-well plates then placed in incubator for 24 h at 32 °C to attach the cells to the bottom of the plate. After 24 or 48 h incubation, the supernatant was slowly collected and discarded. Then 200 μL of PBS was added to each well for washing and then discarded. After that, 50 μL of MTT solution along with 100 μL of complete culture medium (FBS 10% + RPMI-1640) was seeded into the wells. The cells were placed in incubator in 37 °C afterward the adding of MTT solution for peak of 4 h. Afterward eliminating the medium, 200 μL of Methylsulfinylmethane (DMSO) was increased to all well, and an ELISA Plate Reader (Sunrise™, Switzerland) was applied to assess the absorbance at 570.630 nm.

### RNA isolation and qRT-PCR

Following the company’s instruction, TRI Reagent (GeneAll Biotechnology, Seoul, Korea) was utilized for whole RNA extraction from none-transfected (CTRL) negative control (NC) and transfected by anti-miR-21 cells. The extracted RNAs were stored in a − 80 °C freezer until cDNA synthesis. For cDNA synthesis, BIOFACT's RT-PCR Pre Mix synthesis kit made in South Korea was used, to study the expression level of miR-21 and the rate of expression of the target genes. The expression rates of target genes and miR-21 were assessed through qRT-PCR by SYBR™ Green PCR Master Mix (QIAGEN, Hilden, Germany). Glyceraldehyde-3-phosphate dehydrogenase (GAPDH) and U6 were used as an internal control miR-21 functional investigates and confirm the requirement to precisely contemplate the selection of a reference gene in miRNA trials. The relative gene expression were evaluated by the Livak technique^49^. The primers nucleotide sequences are described in Table 2.

$$ \Delta {\text{CT}} = {\text{CT}}_{{{\text{target}}}} {-}{\text{CT}}_{{{\text{reference}}}} . $$

$$ \Delta \Delta {\text{CT}} = \Delta {\text{CT}}_{{{\text{sample}}}} {-}\Delta {\text{CT}}_{{{\text{calibrator}}}} . $$

$$ {\text{Relative }}\;{\text{gene}}\;{\text{ expression}} = {2}^{{ - \, \Delta \Delta {\text{CT}}}} . $$

**Table 2:** The primers nucleotides sequence and miRNA utilized in the qRT-PCR.

Name	Forward/reverse	Sequence
miR-21	Target sequence	5′-UAGCUUAUCAGACUGAUGUUGA-3′
Anti-miR-21	Sequence	5′-UCAACAUCAGUCUGAUAAGCUA-3′
GAPDH	F	AAGGTGAAGGTCGGAGTCAAC
	R	GGGGTCATTGATGGCAACAA
U6	F	CTTCGGCAGCACATATACTAAAATTGG
	R	TCATCCTTGCGCAGGGG
CCL28	F	TGCACGGAGGTTTCACATCAT
	R	TTGGCAGCTTGCACTTTCATC
NR3C2	F	GCCCTGCTGGAATCAACTCT
	R	CGACCTGGAGCCTCGATTTT
SYNPO2	F	ATCAAAGGGCACCGTTGTCT
	R	ACTGCTGCTTGTGGTCCTTT

### Statistical analyses

Several approaches of statistical analysis were used during the course of this study. Differentially expressed genes and WGCNA analysis were identified using the R programming language (Limma and WGCNA packages), and expression value, biomarker capability (ROC curve), and statistical analysis of the aforementioned genes were performed in GraphPad Prism V8, a software for Create publication-quality graphs and analyze your scientific data with t-tests, ANOVA, linear and nonlinear regression, survival analysis and more (https://www.graphpad.com/features). The root-mean-square deviation is used to characterize total quantities (SD). The standard deviation (SD) of a set of variables was used. We used ANOVA, one-way and two-way ANOVA to find the most significant differences across the data sets. GraphPad software was used to do the statistical analysis. A statistically significant difference was found when P 0.05 was found.

### Ethical approval

The study was approved by the ethical committee of Tabriz University of Medical Sciences, Tabriz, Iran.

## Data Availability

All data generated or analyzed in the current research are provided within the manuscript.
